# Circulating Angiopoietin-1 Is Not a Biomarker of Disease Severity or Prognosis in Pulmonary Hypertension

**DOI:** 10.1371/journal.pone.0165982

**Published:** 2016-11-01

**Authors:** Manuel Jonas Richter, Svenja Lena Tiede, Natascha Sommer, Thomas Schmidt, Werner Seeger, Hossein Ardeschir Ghofrani, Ralph Schermuly, Henning Gall

**Affiliations:** 1 Department of Internal Medicine, Justus-Liebig-University Giessen, Universities of Giessen and Marburg Lung Center (UGMLC), Member of the German Center for Lung Research (DZL), Giessen, Germany; 2 Department of Pneumology, Kerckhoff Heart, Rheuma and Thoracic Center, Bad Nauheim, Germany; 3 Department of Medicine, Imperial College London, London, United Kingdom; Vanderbilt University Medical Center, UNITED STATES

## Abstract

**Background:**

Circulating angiopoietin-1 (Ang-1) has been linked to pulmonary hypertension (PH) in experimental studies. However, the clinical relevance of Ang-1 as a biomarker in PH remains unknown. We aimed to investigate the prognostic and clinical significance of Ang-1 in PH using data from the prospectively recruiting Giessen PH Registry.

**Methods:**

Patients with suspected PH (without previous specific pulmonary arterial hypertension [PAH] therapy) who underwent initial right heart catheterization (RHC) in our national referral center between July 2003 and May 2012 and who agreed to optional biomarker analysis were included if they were diagnosed with idiopathic PAH, connective tissue disease-associated PAH (CTD-PAH), PH due to left heart disease (PH-LHD), or chronic thromboembolic PH (CTEPH), or if PH was excluded by RHC (non-PH controls). The association of Ang-1 levels with disease severity (6-minute walk distance and pulmonary hemodynamics) was assessed using linear regression, and the impact of Ang-1 levels on transplant-free survival (primary endpoint) and clinical worsening was assessed using Kaplan—Meier curves, receiver operating characteristic (ROC) analyses, and Cox regression.

**Results:**

151 patients (39, 39, 32, and 41 with idiopathic PAH, CTD-PAH, PH-LHD, and CTEPH, respectively) and 41 non-PH controls were included. Ang-1 levels showed no significant difference between groups (p = 0.8), and no significant associations with disease severity in PH subgroups (p ≥ 0.07). In Kaplan—Meier analyses, Ang-1 levels (stratified by quartile) had no significant impact on transplant-free survival (p ≥ 0.27) or clinical worsening (p ≥ 0.51) in PH subgroups. Regression models found no significant association between Ang-1 levels and outcomes (p ≥ 0.31). ROC analyses found no significant cut-off that would maximize sensitivity and specificity.

**Conclusions:**

Despite a strong pathophysiological association in experimental studies, this first comprehensive analysis of Ang-1 in PH subgroups suggests that Ang-1 is not a predictive and clinically relevant biomarker in PH.

## Introduction

Pulmonary hypertension (PH) is characterized by elevated pulmonary vascular resistance (PVR), reduced cardiac output (CO), and increased pulmonary arterial pressure, due to progressive remodeling of the pulmonary arteries caused by multiple pathophysiological mechanisms [1]. If left untreated, the disease eventually progresses to right heart failure. Patients with PH are classified into five major clinical groups according to current guidelines: pulmonary arterial hypertension (PAH; group 1); pulmonary veno-occlusive disease and/or pulmonary capillary hemangiomatosis (group 1′); persistent PH of the newborn (group 1”); PH due to left heart disease (PH-LHD; group 2); PH due to lung diseases and/or hypoxia (group 3); chronic thromboembolic PH (CTEPH; group 4); and PH with unclear and/or multifactorial mechanisms (group 5) [1]. In the routine treatment and follow-up of patients with PH, a key role has been attributed to parameters and biomarkers associated with long-term survival, time to clinical worsening (TTCW), or disease severity [2, 3]. Numerous important biomarkers such as N-terminal fragment of pro-B-type natriuretic peptide [4], red cell distribution [5], endothelin-1 [6], troponin [7], serum creatinine [8], and glycosylated hemoglobin A1c [9] have been described previously, and N-terminal fragment of pro-B-type natriuretic peptide is recommended as a prognostic biomarker in current guidelines [1]. However, many biomarker studies have focused exclusively on patients with PAH; biomarkers that mirror major pathophysiological pathways and predict long-term outcome in other PH subgroups are still of major interest. In this context, alteration of the angiopoietin—Tie2 ligand—receptor system has been described as a major contributor to endothelial dysfunction in idiopathic PAH (iPAH) [10], congenital heart disease [11], and acute left heart failure [12]. Tie2 is a tyrosine kinase receptor expressed by endothelial cells which is essential for angiogenesis during embryonic cardiovascular development [13–16]. Angiopoietins, comprising a family of growth factors, are ligands of the Tie2 receptor [17]. Major compounds are the Tie2 agonist angiopoietin-1 (Ang-1) [14] and the antagonist angiopoietin-2 (Ang-2) [15]. Ang-1-induced Tie2 phosphorylation leads to activation of endothelial nitric oxide synthase and pathways involved in endothelial permeability, peri-endothelial cell recruitment, and anti-inflammatory signal transduction [17]. During angiogenesis, Ang-1 and the Tie2 receptor are expressed in growing blood vessels [18]. After angiogenesis and lung development, Ang-1 is expressed at a minimally detectable level in the healthy human lung, while the Tie2 receptor remains constitutively expressed and is believed to regulate vascular maintenance [18, 19]. Overexpression of Ang-1 with activation of the Tie2 receptor was found in non-familial PH and CTEPH and was related to disease severity [20, 21]. Moreover, virally-mediated overexpression of Ang-1 in animal studies had a pathological impact on the pulmonary circulation [22, 23]. These studies suggest a role for Ang-1 in the pathological alteration of pulmonary smooth muscle cells in PH [19]. However, other experimental studies found a protective effect of Ang-1 against PAH [24, 25]. Moreover, Ang-1 in the adult vasculature has been suggested to promote vessel integrity [26], inhibit vascular leakage [27], and suppress inflammatory gene expression [28]. The role of Ang-1 in PH therefore remains controversial.

Previously published literature provides little evidence regarding the actual clinical relevance of Ang-1 as a potential biomarker in different subtypes of PH. Ang-1 was investigated as a prognostic biomarker in only one study conducted exclusively in patients with iPAH, in which Ang-1 showed no associations with disease severity or outcome [10]. Therefore, we aimed to clarify the prognostic and clinical relevance of circulating Ang-1 levels and to assess if Ang-1 is related to disease severity in different subtypes of PH. Despite the strong pathophysiological association observed in previous experimental studies, we found no association between Ang-1 levels and disease severity or outcomes in patients with iPAH, CTD-PAH, PH-LHD, or CTEPH enrolled in the Giessen PH Registry. Thus, our results do not support a role for Ang-1 as a predictive and clinically relevant biomarker in these PH subgroups.

## Materials and Methods

### Patients

PAH therapy-naïve patients with suspected PH undergoing initial right heart catheterization (RHC) in our national referral center between July 2003 and May 2012 were included in our study if they agreed to participate in an optional biomarker analysis at the time of registration and had a diagnosis of iPAH, PAH associated with connective tissue disease (CTD-PAH), PH-LHD, or CTEPH assigned by a multidisciplinary board (updated according to current guidelines) [1]. Patients who were assigned a diagnosis of PH with unclear and/or multifactorial mechanisms (PH group 5) or PH due to lung diseases and/or hypoxia (PH group 3) were excluded from the current study. All included patients were enrolled in the single-center Giessen PH Registry, and follow-up data were retrieved from this registry up to April 2015.

The primary outcome was transplant-free survival; patients lost to follow-up were classified as having withdrawn alive on the date of last contact. TTCW was assessed as a secondary endpoint. Clinical worsening was defined as any of the following events (alone or in combination): hospitalization required due to right ventricular failure; escalation of specific PAH therapy; worsening of World Health Organization (WHO) functional class; and/or a decrease in 6-minute walk distance (6MWD) of at least 30 m.

Baseline parameters such as pulmonary hemodynamics, 6MWD, and WHO functional class were collected from all patients as available. Patients with unexplained dyspnea for whom a diagnosis of PH was excluded by RHC were considered as a non-PH control group.

Data collection and analyses were approved by the ethics committee of the Faculty of Medicine at the University of Giessen (Approval No. 100/2013). The investigation conforms with the principles outlined in the Declaration of Helsinki [29]. Each patient gave written, informed consent for the use and storage of plasma for future biomarker analyses on the day of sample collection.

### Blood sampling and laboratory analyses of angiopoietin-1

All blood samples were collected as central venous samples at the time of the initial RHC and before the initiation of any PAH-targeted therapy. All samples were immediately placed on ice and then centrifuged at 3000 ×g for 10 min within 45 min after collection. Blood samples were divided into aliquots and stored at –80°C. Ang-1 levels were measured in-house using the Human Angiopoietin-1 Quantikine^®^ enzyme-linked immunosorbent assay (ELISA) from R&D Systems Inc., Minneapolis, USA. The assay has a sensitivity of 10.3 pg/mL and an assay range of 62.5–4000 pg/mL. Samples were diluted 15fold, as instructed by the manufacturer of the kit. The investigator who determined Ang-1 levels was unaware of each patient’s baseline parameters.

### Statistical analysis

Data are presented as mean ± standard deviation or median [interquartile range (IQR)] for normally or non-normally distributed parameters, respectively. To test for differences between groups, the Pearson chi-square test, one-way ANOVA, or Kruskal—Wallis test was used as appropriate. In further statistical analyses involving Ang-1 levels, natural log-transformed concentrations were used, unless stated otherwise. Association of Ang-1 levels with disease severity at baseline (6MWD and pulmonary hemodynamics) was assessed using linear regression analysis.

The prognostic relevance of baseline parameters for transplant-free survival and for TTCW was assessed using separate univariate Cox proportional-hazards regression models. All baseline parameters were assessed as continuous variables and p < 0.1 was considered statistically significant in the univariate analyses.

Moreover, the ability of Ang-1 levels (divided into quartiles) to predict overall survival or TTCW was assessed using Kaplan—Meier analyses with log-rank tests. In addition, receiver operating characteristic (ROC) analyses were performed to assess cut-offs of Ang-1 levels. Statistical analysis was performed using SPSS, version 21.0 (IBM, Armonk, New York, USA).

## Results

### Baseline characteristics

In total, the study included 151 patients with PH. The PH classification was iPAH in 39 patients, CTD-PAH in 39 patients, PH-LHD in 32 patients, and CTEPH in 41 patients.

Baseline characteristics are shown in Table 1. Patients with iPAH were mainly female and in WHO functional class III with a mean 6MWD of 350.9 m (Table 1). They had severely impaired pulmonary hemodynamics with reduced cardiac index and elevated PVR and mean pulmonary arterial pressure (mPAP) compared with non-PH controls. Patients with CTD-PAH had a higher proportion of women and patients in WHO functional class IV and a lower 6MWD than patients with iPAH. Pulmonary hemodynamics differed slightly with a lower mPAP and PVR and higher cardiac index in the CTD-PAH group compared with the iPAH group. Patients with PH-LHD were significantly older than the other groups and were mostly in WHO functional class II and III, with a severely reduced 6MWD. Pulmonary hemodynamics showed the characteristic pattern of post-capillary PH with significant elevation of pulmonary arterial wedge pressure (PAWP) and mPAP accompanied by a preserved cardiac index and slightly elevated PVR. The majority of patients with CTEPH presented in WHO functional class III with a pre-capillary PH pattern comprising an elevated mPAP and PVR, a reduced cardiac index, and a PAWP within the normal range. In the CTEPH group, 6MWD was higher than in the CTD-PAH or PH-LHD groups, but lower than in the iPAH group. The non-PH control group was broadly matched to the PH study population in terms of sex and WHO functional class, comprising mainly female patients in WHO functional class III. Although the non-PH control group had a low 6MWD (327.4 m), the pulmonary hemodynamic profile was within the normal range. Almost 40% of the non-PH controls had cardiac disorders such as valvular disease, but PAWP values did not indicate left ventricular dysfunction. Ang-1 levels showed no significant difference between PH subgroups and non-PH controls, although there was a trend towards higher levels in controls and lower levels in patients with iPAH (Table 1).

**Table 1:** Baseline characteristics.

Patient population	Patients with PH				Non-PH controls	p value
PH subtype	iPAH	CTD-PAH	PH-LHD	CTEPH		
Patients, n	39	39	32	41	41	
Male/Female (%)	38.5/61.5	20.5/79.5	37.5/62.5	53.7/46.3	36.6/63.4	0.05^a^
Age (years)	54.4 ± 15.7	60.3 ± 13.9	73.1 ± 9.8	65.9 ± 12.5	66.1 ± 11.5	<0.001^b^
WHO functional class (%)						0.01^a^
II	33.3	15.8	38.7	27.5	21.4	
III	56.4	55.3	54.8	62.5	71.4	
IV	10.3	28.9	6.5	10.0	7.1	
RHC						
mPAP (mm Hg)	47.2 ± 15.0	43.6 ± 10.8	38.6 ± 9.6	40.7 ± 10.4	18.1 ± 3.8	<0.001^b^
PVR (dyne*s/cm^5^)	725.0 [527.0]	670.0 [497.0]	313.0 [261.8]	590.0 [425.0]	150.0 [107.5]	<0.001^c^
Cardiac index (L/min/m^2^)	2.3 ± 0.6	2.4 ± 0.5	2.6 ± 0.6	2.3 ± 0.6	2.7 ± 0.7	<0.001^b^
CO (L/min)	4.1 ± 1.0	4.2 ± 1.0	4.9 ± 1.2	4.4 ± 1.3	5.1 ± 1.3	<0.001^b^
PAWP (mm Hg)	7.7 ± 3.2	7.5 ± 3.6	17.6 ± 6.3	9.4 ± 4.0	8.4 ± 3.6	<0.001^b^
6MWD (m)	350.9 ± 131.2	307.1 ± 134.1	299.1 ± 97.1	321.3 ± 100.6	327.4 ± 128.9	0.5^b^
Ang-1 (pg/mL)	2849.3 [3981.5]	3130.9 [6644.8]	3759.2 [4315.6]	3525.1 [4226.1]	3907.9 [3706.0]	0.8^c^

Values represent mean ± standard deviation or median [interquartile range].

6MWD, six-minute walk distance; Ang-1, angiopoietin-1; CO, cardiac output; CTD, connective tissue disease; CTEPH, chronic thromboembolic pulmonary hypertension; iPAH, idiopathic pulmonary arterial hypertension; LHD, left heart disease; PAH, pulmonary arterial hypertension; mPAP, mean pulmonary arterial pressure; PAWP, pulmonary arterial wedge pressure; PH, pulmonary hypertension; PVR, pulmonary vascular resistance; RHC, right heart catheterization; WHO, World Health Organization.

^a^Pearson chi-square test;

^b^one-way ANOVA;

^c^Kruskal—Wallis test.

### Association of angiopoietin-1 with disease severity

**Fig 1:**
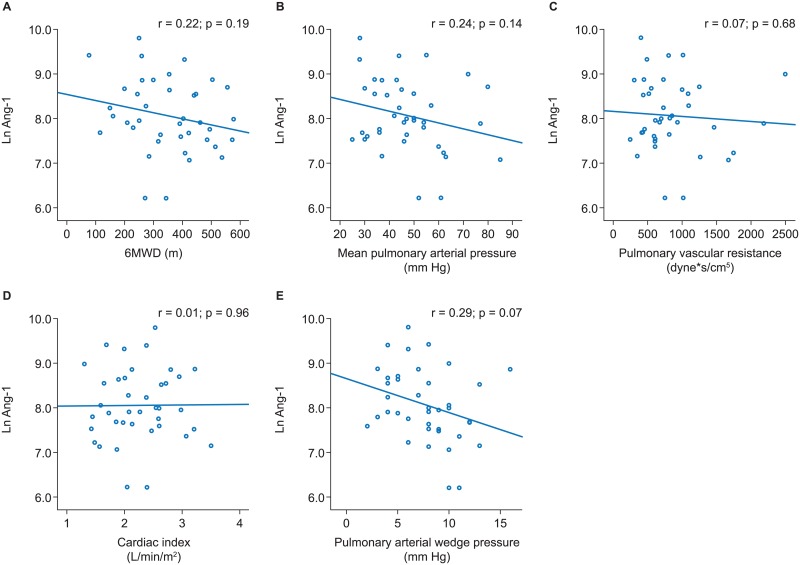
Associations of angiopoietin-1 with 6MWD and pulmonary hemodynamics in iPAH. Scatter plots and results of linear regression analyses are shown for 6MWD (A), mean pulmonary arterial pressure (B), pulmonary vascular resistance (C), cardiac index (D), and pulmonary arterial wedge pressure (E). 6MWD, 6-minute walk distance; iPAH, idiopathic pulmonary arterial hypertension.

**Fig 2:**
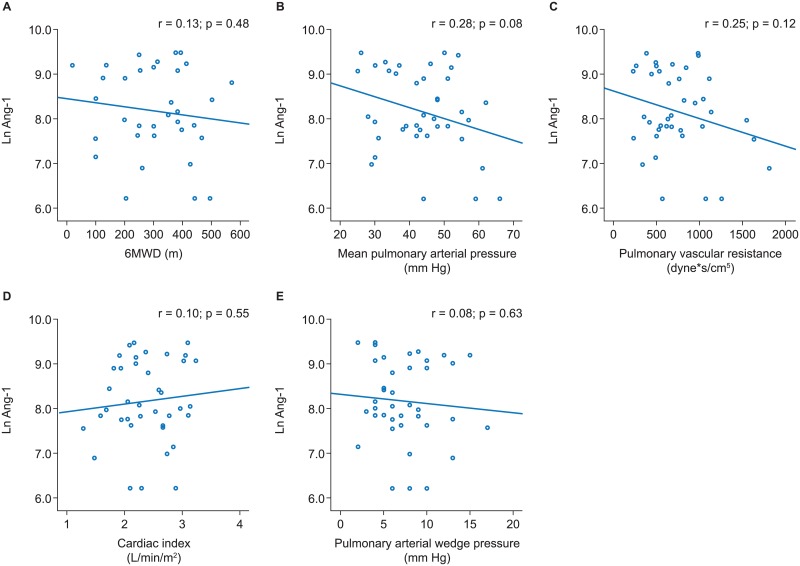
Associations of angiopoietin-1 with 6MWD and pulmonary hemodynamics in CTD-PAH. Scatter plots and results of linear regression analyses are shown for 6MWD (A), mean pulmonary arterial pressure (B), pulmonary vascular resistance (C), cardiac index (D), and pulmonary arterial wedge pressure (E). 6MWD, 6-minute walk distance; CTD-PAH, connective tissue disease-associated pulmonary arterial hypertension.

**Fig 3:**
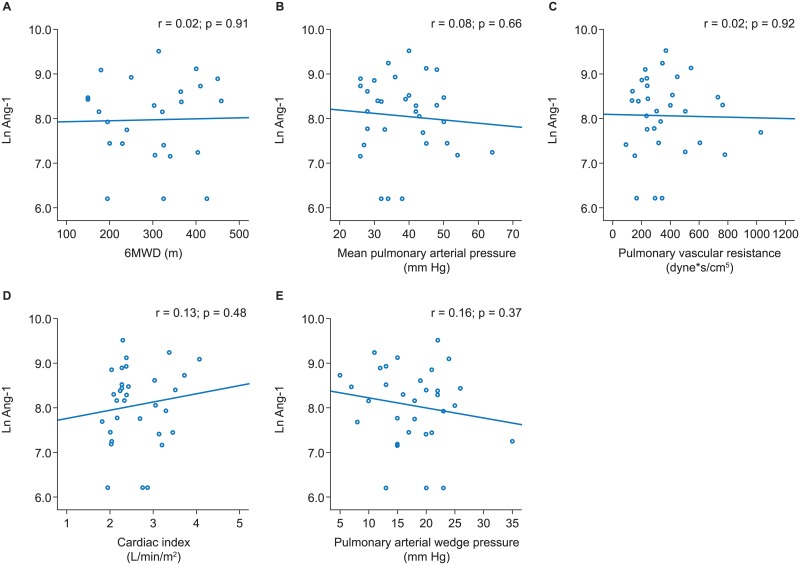
Associations of angiopoietin-1 with 6MWD and pulmonary hemodynamics in PH-LHD. Scatter plots and results of linear regression analyses are shown for 6MWD (A), mean pulmonary arterial pressure (B), pulmonary vascular resistance (C), cardiac index (D), and pulmonary arterial wedge pressure (E). 6MWD, 6-minute walk distance; PH-LHD, pulmonary hypertension due to left heart disease.

**Fig 4:**
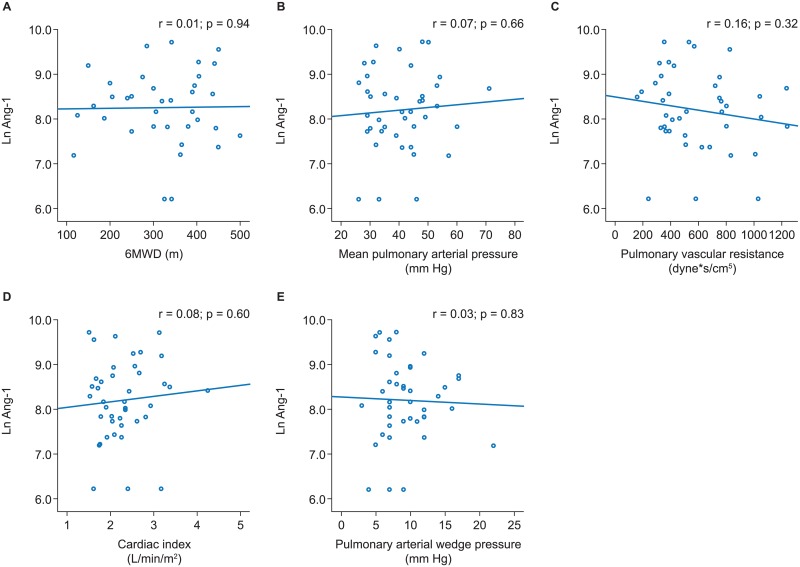
Associations of angiopoietin-1 with 6MWD and pulmonary hemodynamics in CTEPH. Scatter plots and results of linear regression analyses are shown for 6MWD (A), mean pulmonary arterial pressure (B), pulmonary vascular resistance (C), cardiac index (D), and pulmonary arterial wedge pressure (E). 6MWD, 6-minute walk distance; CTEPH, chronic thromboembolic pulmonary hypertension.

Across all PH subgroups no significant associations of Ang-1 with 6MWD or pulmonary hemodynamics were evident (Figs 1–4).

**Fig 5:**
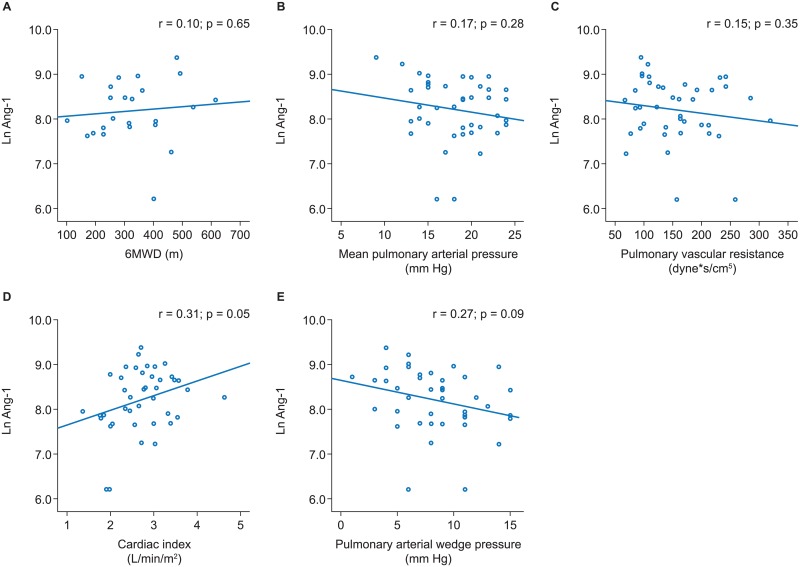
Associations of angiopoietin-1 with 6MWD and pulmonary hemodynamics in non-PH controls. Scatter plots and results of linear regression analyses are shown for 6MWD (A), mean pulmonary arterial pressure (B), pulmonary vascular resistance (C), cardiac index (D), and pulmonary arterial wedge pressure (E). 6MWD, 6-minute walk distance; PH, pulmonary hypertension.

Non-PH controls showed a borderline significant association of Ang-1 with cardiac index (r = 0.307, p = 0.051), but no significant associations with other baseline parameters were evident (Fig 5).

### Primary and secondary endpoint analysis: transplant-free survival and clinical worsening

In the total PH cohort, 57 (29.7%) of the patients died during a mean follow-up period of 31.5 ± 40.8 months (median [IQR]: 32 [44] months), and overall survival was 89.4% at 1 year and 67.2% at 3 years. When assessed by PH subtype, 1-and 3- year overall survival was 94.8% and 77.9%, respectively, in the subgroup with iPAH, 78.9% and 52.6%, respectively, in patients with CTD-PAH, 95.7% and 50.3%, respectively, in those with PH-LHD, and 88.7% and 75.6%, respectively, in those with CTEPH.

In the total PH cohort, the mean TTCW was 25.0 ± 31.1 months (median [IQR]: 18 [38] months), with 101 (52.6%) patients experiencing a clinical worsening event. The cumulative proportion of patients experiencing a clinical worsening event was 28.8% at 1 year and 67.3% at 3 years. When assessed by PH subtype, the cumulative proportion of patients experiencing a clinical worsening event at 1 year and 3 years was 7.0% and 14.5%, respectively, in the subgroup with iPAH, 5.0% and 32.7%, respectively, in patients with CTD-PAH, 28.3% and 100%, respectively, in those with PH-LHD, and 18.8% and 90.5%, respectively, in those with CTEPH.

#### Impact of angiopoietin-1 as a prognostic biomarker

**Fig 6:**
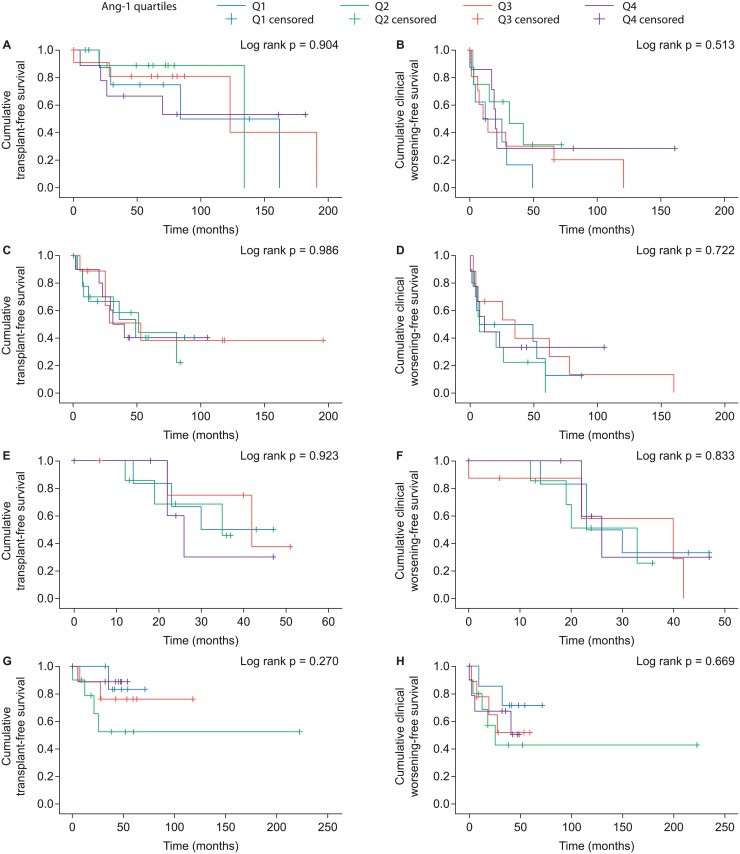
Kaplan—Meier survival and time to clinical worsening curves stratified by angiopoietin-1 quartile. Analyses were conducted separately in patients with idiopathic pulmonary arterial hypertension (A–B), connective tissue disease-associated pulmonary arterial hypertension (C–D), pulmonary hypertension due to left heart disease (E–F), and chronic thromboembolic pulmonary hypertension (G–H).

Kaplan—Meier analyses of outcomes stratified by Ang-1 quartile revealed no significant impact of Ang-1 levels on overall survival and TTCW in any of the studied PH subgroups (Fig 6; see S1 Table for quartile thresholds). In addition, ROC analyses in the different PH subgroups failed to detect a relevant cut-off value for circulating Ang-1 that would maximize sensitivity and specificity (S2 and S3 Tables).

The results of univariate regression models also did not support a role for Ang-1 as a prognostic biomarker in the studied PH subgroups. Ang-1 levels were not associated with transplant-free survival or TTCW in patients with iPAH, PAH-CTD, PH-LHD, or CTEPH in the univariate regression models (Tables 2 and 3). Instead, the following parameters were significantly associated with mortality/transplant in the models: WHO functional class IV and 6MWD in patients with iPAH; age, WHO functional class IV, cardiac index, CO, and 6MWD in patients with CTD-PAH; PVR, cardiac index, CO, and PAWP in patients with PH-LHD; and gender in patients with CTEPH (Table 2). Parameters significantly associated with TTCW in the models were as follows: WHO functional class IV in patients with iPAH; WHO functional class IV, mPAP, PVR, cardiac index, CO, and 6MWD in patients with CTD-PAH; cardiac index, CO, and 6MWD in patients with PH-LHD; and gender, age, WHO functional class IV, and mPAP in patients with CTEPH (Table 3). As Ang-1 levels showed no significant association with outcomes in the univariate regression models, a multivariate model was not constructed.

**Table 2:** Angiopoietin-1 levels and other baseline parameters as predictors of overall mortality/transplant in patients with different PH subtypes.

Patient population								
PH subtype	iPAH		CTD-PAH		PH-LHD		CTEPH	
	HR (95% CI)^a^	p value	HR (95% CI)^a^	p value	HR (95% CI)^a^	p value	HR (95% CI)^a^	p value
Gender (male vs female)	2.02 [0.67, 6.22]	0.22	1.71 [0.69, 4.21]	0.24	2.02 [0.6.93]60,	0.26	8.46 [1.04, 69.09]	0.05
Age (years)	1.02 [0.98, 1.06]	0.32	1.03 [1.00, 1.07]	0.09	0.98 [0.93, 1.04]	0.57	0.98 [0.93, 1.02]	0.31
WHO functional class								
II	Reference		Reference		Reference		Reference	
III vs II	3.08 [0.64, 14.87]	0.16	0.85 [0.23, 3.16]	0.81	1.87 [0.48, 7.25]	0.36	0.67 [0.11, 4.00]	0.66
IV vs II	25.34 [3.58, 179.48]	0.001	5.01 [1.25, 20.06]	0.02	1.66 [0.17, 16.2]	0.67	3.03 [0.42, 21.64]	0.27
mPAP (mm Hg)	0.98 [0.94, 1.03]	0.47	1.01 [0.98, 1.05]	0.47	1.02 [0.95, 1.09]	0.67	1.01 [0.95, 1.08]	0.66
PVR (dyne*s/cm^5^)	1.00 [1.00, 1.00]	0.37	1.01 [1.00, 1.02]	0.12	1.00 [0.99, 1.00]	0.06	1.00 [1.00, 1.01]	0.80
Cardiac index (L/min/m^2^)	0.77 [0.30, 1.96]	0.58	3.23 [1.25, 8.33]	0.02	3.69 [1.44, 9.45]	0.007	0.51 [0.12, 2.16]	0.36
CO (L/min)	1.05 [0.67, 1.65]	0.82	0.50 [0.32, 0.80]	0.003	1.78 [1.17, 2.70]	0.007	0.75 [0.36, 1.58]	0.45
PAWP (mm Hg)	1.06 [0.89, 1.26]	0.52	1.05 [0.94, 1.17]	0.39	1.09 [1.00, 1.18]	0.06	0.94 [0.77, 1.15]	0.54
6MWD (m)	0.99 [0.98, 0.99]	0.001	0.99 [0.99, 1.00]	< 0.001	1.00 [0.99, 1.00]	0.29	1.01 [0.99, 1.01]	0.82
Ln Ang-1 (pg/mL)	1.26 [0.60, 2.63]	0.55	1.30 [0.79, 2.10]	0.31	1.18 [0.65, 2.17]	0.58	0.90 [0.39, 2.11]	0.81

6MWD, six-minute walk distance; Ang-1, angiopoietin-1; CI, confidence interval; CO, cardiac output; CTD, connective tissue disease; CTEPH, chronic thromboembolic pulmonary hypertension; HR, hazard ratio; iPAH, idiopathic pulmonary arterial hypertension; LHD, left heart disease; Ln, natural log; PAH, pulmonary arterial hypertension; mPAP, mean pulmonary arterial pressure; PAWP, pulmonary arterial wedge pressure; PH, pulmonary hypertension; PVR, pulmonary vascular resistance; WHO, World Health Organization.

^a^HR and 95% CI values were calculated by univariate Cox regression analysis.

**Table 3:** Angiopoetin-1 levels and baseline parameters as predictors of clinical worsening according to patient population.

Patient population								
PH subtype	iPAH		CTD-PAH		PH-LHD		CTEPH	
	HR (95% CI)^a^	p value	HR (95% CI)^a^	p value	HR (95% CI)^a^	p value	HR (95% CI)^a^	p value
Gender (male vs female)	2.25 [0.63, 8.03]	0.21	1.80 [0.73, 4.44]	0.20	2.75 [0.80, 9.45]	0.11	7.17 [0.88, 58.44]	0.07
Age (years)	1.00 [0.97, 1.03]	0.77	1.01 [0.99, 1.04]	0.38	0.98 [0.94, 1.02]	0.34	0.95 [0.92, 0.99]	0.006
WHO functional class								
II	Reference		Reference		Reference		Reference	
III vs II	1.45 [0.59, 3.55]	0.41	0.66 [0.23, 1.92]	0.45	1.58 [0.53, 4.76]	0.42	2.55 [0.55, 11.84]	0.23
IV vs II	38.42 [5.47, 269.82]	0.001	2.89 [0.93, 8.98]	0.07	0.82 [0.09, 7.22]	0.86	7.07 [1.15, 43.49]	0.04
mPAP (mm Hg)	1.01 [0.99, 1.04]	0.41	1.03 [1.00, 1.06]	0.07	1.05 [0.99, 1.11]	0.12	1.04 [1.00, 1.09]	0.07
PVR (dyne*s/cm^5^)	1.00 [1.00, 1.01]	0.25	1.00 [1.00, 1.02]	0.02	1.00 [1.00, 1.01]	0.95	1.00 [1.00, 1.03]	0.31
Cardiac index (L/min/m^2^)	1.81 [0.87, 3.85]	0.11	4 [1.71, 9.43]	0.001	2.20 [0.94, 5.12]	0.07	1.77 [0.63, 4.95]	0.28
CO (L/min)	1.27 [0.85, 1.89]	0.24	2.04 [1.35, 3.13]	0.001	1.44 [0.98, 2.10]	0.06	1.00 [0.65, 1.54]	0.99
PAWP (mm Hg)	1.07 [0.95, 1.21]	0.28	1.02 [0.92, 1.13]	0.73	1.02 [0.94, 1.11]	0.70	1.01 [0.90, 1.14]	0.86
6MWD (m)	0.99 [0.99, 1.01]	0.13	1.00 [0.99, 1.00]	0.002	0.99 [0.99, 1.00]	0.03	1.00 [0.99, 1.01]	0.82
Ln Ang-1 (pg/mL)	0.90 [0.54, 1.51]	0.68	1.00 [0.67, 1.49]	0.99	1.16 [0.72, 1.88]	0.55	1.18 [0.64, 2.19]	0.60

Ang-1, angiopoietin-1; CI, confidence interval; CO, cardiac output; CTD, connective tissue disease; CTEPH, chronic thromboembolic pulmonary hypertension; HR, hazard ratio; iPAH, idiopathic pulmonary arterial hypertension; LHD, left heart disease; Ln: natural log; PAH, pulmonary arterial hypertension; mPAP, mean pulmonary arterial pressure; PAWP, pulmonary arterial wedge pressure; PH, pulmonary hypertension; PVR, pulmonary vascular resistance; WHO, World Health Organization.

^a^HR and 95% CI values were calculated by univariate Cox regression analysis.

## Discussion

To the best of our knowledge, this is the first study to evaluate the potential of Ang-1 as a biomarker in patients with different PH subtypes (iPAH, CTD-PAH, PH-LHD, and CTEPH). The novel findings of our study include the following: 1) Ang-1 levels were not associated with transplant-free survival; 2) Ang-1 levels were also not associated with TTCW; and 3) Ang-1 levels were not associated with disease severity or functional capacity. Thus, circulating Ang-1 failed as a prognostic and clinically relevant biomarker in patients with PH.

Patients with iPAH in our study population showed a characteristic precapillary PH pattern, impaired WHO functional class, and exercise capacity broadly similar to baseline demographics of previous studies of patients with iPAH [10, 30, 31]. Patients with CTD-PAH also showed a demographic pattern broadly comparable to that observed in previous cohorts [32, 33]. Patients with PH-LHD showed a predominately post-capillary PH pattern with a slightly elevated PVR and a substantially reduced exercise capacity as assessed by 6MWD. The hemodynamic parameters and exercise capacity of our cohort with CTEPH mirrored the typical findings of this patient population with a substantial impairment of right ventricular function [34, 35]. The non-PH control group showed a pulmonary hemodynamic profile within the normal range and did not have left ventricular dysfunction; nevertheless, patients in this group had dyspnea and a high prevalence of cardiac morbidity, and were mainly in WHO functional class III with a reduced exercise capacity.

In our study, median circulating Ang-1 levels showed no statistically significant difference between the various PH subgroups and the non-PH control group. A previous analysis using a capture antibody within an ELISA system showed median circulating Ang-1 levels of 4000 pg/mL and 8000 pg/mL in female and male controls, respectively, comparable to our data [36]. However, another study using a commercially available ELISA reported median circulating Ang-1 levels of 10 000 pg/ml in healthy controls [37]. Methodological differences may lead to differences in reported levels of Ang-1 between studies, which must be considered when comparing results. Nevertheless, these ELISA data suggest that the lack of detectable upregulation of circulating Ang-1 in our cohort with PH is unlikely to be due to an unusually high level of circulating Ang-1 in our control group. It should be noted that previous studies which reported Ang-1 overexpression in patients with PH were based on analysis of lung tissue (by western blot and reverse-transcriptase polymerase chain reaction [RT-PCR]) [20, 21] and did not detect Ang-1 in the patients’ peripheral blood by RT-PCR [21].

We chose to assess the clinical relevance of Ang-1 in PH because this is a controversial field in need of further research; previous studies pointed towards an important function for the Ang-1–Tie2 pathway in PH, but with conflicting results regarding the nature of that function (pathological or protective) and which pathway components are altered by the disease (Ang-1 or Tie2). Experimental studies linked overexpression of Ang-1 and increased Tie-2 phosphorylation to medial thickening in pulmonary arterioles, resulting in obstruction of small pulmonary blood vessels [22, 23]. Moreover, Ang-1 overexpression was associated with the release of growth factors and serotonin, resulting in stimulation of smooth muscle cell hyperplasia [22]. A study of lung tissue from people with different forms of PH showed that Ang-1 was overexpressed and linked to exaggerated pulmonary smooth muscle cell hyperplasia and downregulation of the bone morphogenetic protein receptor (BMPR) 1A, a component of the BMPR complex which is also inactivated in hereditary PAH (by mutation of BMPR2) [20]. Pulmonary Ang-1 expression was also significantly related to disease severity in CTEPH [21]. However, another in vitro study showed that Ang-1 expression in lung tissue and cultured pulmonary smooth muscle cells did not differ between patients with iPAH and controls; instead, the Tie-2 receptor was overexpressed in pulmonary endothelial cells and mediated the pathological effects of Ang-1 by stimulating synthesis of endothelium-derived growth factors [38]. Conversely, an animal model of PH displayed a reduced expression of the Tie-2 receptor accompanied by increased endothelial cell apoptosis; in this model, cell-based gene transfer of Ang-1 restored Tie-2 expression, reduced endothelial cell apoptosis, and inhibited the development of PH, leading the authors to suggest that the previously reported overexpression of Ang-1 in PH may have been a compensatory response to vascular damage [39].

In our cohort of patients with iPAH, Ang-1 failed to show an association with disease severity and failed to predict transplant-free survival or TTCW. These findings are in accordance with results from Kumpers and coworkers who assessed circulating Ang-1 levels exclusively in patients with iPAH and failed to demonstrate an association with disease severity and long-term survival [10]. Interestingly, our data showed for the first time that Ang-1 was also not associated with disease severity, long-term transplant-free survival, and TTCW in other PH subtypes such as CTD-PAH, PH-LHD, and CTEPH. Furthermore, our data support the hypothesis that circulating Ang-1 plays an inferior role within the pathological alteration of the Ang/Tie-2 system in the actual clinical setting of PH. Data from other studies in patients with acute coronary syndrome [40] and other cardiovascular disease [41] indicated that the clinical impact of circulating Ang-1 –in contrast to other angiopoietins—is probably small. One can speculate that although the pathophysiological association of Ang-1 with PH has a strong basis in experimental studies, circulating Ang-1 levels are not capable of mirroring clinical PH-specific features. By contrast, Ang-2 was significantly related to disease severity and long-term outcome in various cardiovascular diseases including iPAH and was identified as a clinically relevant biomarker [10–12, 42–44]. We speculate that this discrepancy might be attributed to the different release mechanisms of both angiopoietins and their modes of action within the pulmonary circulation. Ang-2 is stored in the endothelium and is rapidly released in response to different cellular/molecular activators (including inflammatory cytokines, activated platelets, and leukocytes), changes in blood flow, and hypoxemia [17, 45], which comprise hallmark features of endothelial dysfunction in PAH. Therefore, circulating Ang-2 might reflect disease activity and severity in PH more accurately than circulating Ang-1. In contrast to Ang-2, several studies have shown that hypoxia itself does not stimulate the expression of Ang-1 [46, 47]. Moreover, Ang-1 is produced constitutively by endothelial cells at considerable levels, resulting in comparably low circulating concentrations, without known transcriptional activators within the pulmonary circulation [17]. Thus, while Ang-1 may be locally upregulated in the lung tissue of patients with PH [20, 21], high levels of Ang-2 may be released under pathological conditions such as PH, and the release of Ang-2 can be promoted by factors that also induce Ang-2 expression, such as hypoxia or increased vascular endothelial growth factor levels. We acknowledge that in animal models or in vitro studies certain pathophysiological features of PH can be attributed to locally produced Ang-1; however, as PH progresses in adult patients, other angiopoietins and growth factors [48] might take center stage and be released into the circulation.

This study is limited by its single-center design, the sample size and the lack of a healthy control group. Furthermore, we were unable to differentiate conclusively between PH-related and non-PH-related deaths in a substantial proportion of the patients with PH. Moreover, due to the observational study design we were not able to draw direct associations between Ang-1 levels and the precise impact on endothelial or vascular smooth muscle cells on a molecular level. In addition, Ang-1 is not exclusively expressed within the pulmonary vasculature [49] and presents with an altered expression pattern in cases of severe chronic renal disease or various liver disorders [50–54]. Therefore, concomitant chronic renal or liver disease might have influenced Ang-1 levels in our PH groups and affected the baseline comparison. Moreover, we were unable to rule out concomitant liver or renal disease within the non-PH controls. The pivotal study of angiopoietins by Kumpers and coworkers also did not provide information on the prevalence of liver or renal disease [10]. Nevertheless, our non-PH control group represents typical patients with PH-like symptoms for whom a diagnostic separation by a biomarker would be helpful. The involvement of Ang-1 in liver and kidney diseases, common comorbidities in these patients, might prevent Ang-1 from being a useful biomarker in these patients. However, as current PH guidelines [1] demand exclusion of concomitant very severe liver and renal diseases for the diagnosis of idiopathic PAH and CTD-PAH, the influence of renal/liver disease on Ang-1 levels within these PH groups might be small and questionable.

## Conclusions

In conclusion, this is the first study to investigate Ang-1 as a clinically relevant prognostic biomarker in PH. In iPAH, CTD-PAH, PH-LHD, and CTEPH, circulating Ang-1 levels were not associated with transplant-free survival, TTCW, or disease severity. Our data indicate that, in contrast to other components of the Ang/Tie2 system, circulating Ang-1 plays a clinically inferior role in PH.

## Supporting Information

S1 Table Angiopoietin-1 quartile thresholds for each PH subgroup in the study cohort. (DOCX)

S2 Table ROC analyses of angiopoietin-1 concentration as a predictor of transplant-free survival. (DOCX)

S3 Table ROC analyses of angiopoietin-1 concentration as a predictor of clinical worsening. (DOCX)
