# Supplementary material for: Circulating Angiopoietin-1 Is Not a Biomarker of Disease Severity or Prognosis in Pulmonary Hypertension
Source: PLoS One. 2016 Nov 1;11(11):e0165982. doi: 10.1371/journal.pone.0165982 (PMC5089726; doi:10.1371/journal.pone.0165982)
Supplement: S1 Table — (DOCX) [file pone.0165982.s001.docx]

**Supporting Information**

**Circulating Angiopoietin-1 is Not a Biomarker of Disease Severity or Prognosis in Pulmonary Hypertension**

Manuel Jonas Richter, Svenja Lena Tiede, Natascha Sommer, Thomas Schmidt, Werner Seeger, Hossein Ardeschir Ghofrani, Ralph Schermuly and Henning Gall

**S1 Table. Angiopoietin-1 quartile thresholds for each PH subgroup in the study cohort.**

|  | **Angiopoietin-1 quartile thresholds (pg/mL)** | | | |
| --- | --- | --- | --- | --- |
| **PH subgroup** | **Q1** | **Q2** | **Q3** | **Q4** |
| iPAH (n = 39) | ≤ 1856.3 | 1856.3–2849.3 | 2849.3–5837.8 | ≥ 5837.8 |
| CTD-PAH (n = 39) | ≤ 2045.5 | 2045.5–3130.9 | 3130.9–8690.3 | ≥ 8690.3 |
| PH-LHD (n = 32) | ≤ 1722.8 | 1722.8–3759.2 | 3759.2–6038.4 | ≥ 6038.4 |
| CTEPH (n = 41) | ≤ 2277.1 | 2277.1–3525.1 | 3525.1–6503.1 | ≥ 6503.1 |

CTD-PAH, connective tissue disease-associated pulmonary arterial hypertension; CTEPH, chronic thromboembolic pulmonary hypertension; iPAH, idiopathic pulmonary arterial hypertension; PH, pulmonary hypertension; PH-LHD, pulmonary hypertension due to left heart disease.
