# Supplementary material for: Circulating Angiopoietin-1 Is Not a Biomarker of Disease Severity or Prognosis in Pulmonary Hypertension
Source: PLoS One. 2016 Nov 1;11(11):e0165982. doi: 10.1371/journal.pone.0165982 (PMC5089726; doi:10.1371/journal.pone.0165982)
Supplement: S2 Table — (DOCX) [file pone.0165982.s002.docx]

**Supporting Information**

**Circulating Angiopoietin-1 is Not a Biomarker of Disease Severity or Prognosis in Pulmonary Hypertension**

Manuel Jonas Richter, Svenja Lena Tiede, Natascha Sommer, Thomas Schmidt, Werner Seeger, Hossein Ardeschir Ghofrani, Ralph Schermuly and Henning Gall

**S2 Table. ROC analyses of angiopoietin-1 concentration as a predictor of transplant-free survival.**

| **PH subtype** | **AUC** | **95% CI** | **p value** | **Comment** |
| --- | --- | --- | --- | --- |
| iPAH | 0.61 | 0.32, 0.90 | 0.42 | No significant cut-off found that maximized sensitivity and specificity |
| CTD-PAH | 0.51 | 0.28, 0.75 | 0.91 | No significant cut-off found that maximized sensitivity and specificity |
| PH-LHD | – | – | – | ROC analyses did not converge |
| CTEPH | 0.53 | 0.17, 0.89 | 0.86 | No significant cut-off found that maximized sensitivity and specificity |

AUC, area under the curve; CI, confidence interval; CTD-PAH, connective tissue disease-associated pulmonary arterial hypertension; CTEPH, chronic thromboembolic pulmonary hypertension; iPAH, idiopathic pulmonary arterial hypertension; PH, pulmonary hypertension; PH-LHD, pulmonary hypertension due to left heart disease; ROC, receiver operating characteristic.
